# Diet-Derived Antioxidants and Risk of Kidney Stone Disease: Results From the NHANES 2007–2018 and Mendelian Randomization Study

**DOI:** 10.3389/fnut.2021.738302

**Published:** 2021-12-21

**Authors:** Zhongyu Jian, Menghua Wang, Xi Jin, Hong Li, Kunjie Wang

**Affiliations:** ^1^Department of Urology, Institute of Urology (Laboratory of Reconstructive Urology), West China Hospital, Sichuan University, Chengdu, China; ^2^West China Biomedical Big Data Center, Sichuan University, Chengdu, China

**Keywords:** Mendelian randomization, kidney stone disease, nephrolithiasis, antioxidants, oxidative stress

## Abstract

We aimed to explore the associations between diet-derived antioxidants and kidney stone disease (KSD) risk in this study. We performed weighted multivariable-adjusted logistic regression to assess the associations between the six main diet-derived antioxidants and the risk of KSD by using data from the National Health and Nutrition Examination Survey (NHANES) 2007–2018. Then, we used the Mendelian randomization (MR) approach to verify the causal relationships between circulating antioxidants levels and KSD risk. Genetic tools were extracted from published genome-wide association studies (GWAS). Summary data for KSD was from the FinnGen study and UK biobank. Inverse variance weighted (IVW) was the primary analysis. The 26,438 participants, including 2,543 stone formers, were included for analyses. There were no significant associations between retinol, vitamin B6, vitamin C, vitamin E, and lycopene intake with the risk of KSD across all the quartile categories. Similarly, pooled odds ratio (OR) for KSD risk in genetically predicted per unit change were 1.25 (95% CI: 0.39, 4.02; *p* = 0.712), 1.14 (95% CI: 0.84, 1.53; *p* = 0.400), 0.75 (95% CI: 0.52, 1.10; *p* = 0.141), 1.66 (95% CI: 0.80, 3.46; *p* = 0.178), 1.27 (95% CI: 0.29, 5.62; *p* = 0.756), and 0.92 (95% CI: 0.76, 1.12; *p* = 0.417) for retinol, β-carotene, vitamin B6, vitamin C, α-tocopherol, and lycopene, respectively. The above estimates were replicated in the secondary analyses using UK biobank data. Our study did not support a causal association between circulating antioxidants levels and KSD risk. However, these findings should be verified in larger sample-size MR due to the pleiotropy and other limitations.

## Introduction

Kidney stone disease (KSD) affects ~9% of the USA population and is increasingly prevalent and becoming costlier (1, 2). It is estimated that the annual cost exceeded $5 billion in the USA in 2005 (3) and will further increase by 1.24 billion by 2030 with the increase in obesity, diabetes, and other risk factors for KSD (4, 5). About one-third of patients with an initial episode will have a stone recurrence (6), and the risk will be higher in patients with existing stone recurrence (7). Besides, patients with KSD are already at risk of renal impairment (8) and cardiovascular diseases (9). Although KSD is a common disease with a high recurrence worldwide, the pathophysiology is not well-understood, which leads to a poor prevention strategy.

In animal and *in vitro* studies, oxidative stress has been proposed as an essential component in the development and progression of calcium oxalate stone by promoting kidney damage (10–13). An analysis of clinical data suggests that antioxidant deficits are common in patients with idiopathic recurrent calcium stones (14). Serum levels of some antioxidants, including β-carotene, are lower in participants with a self-reported history of KSD (15). Consequently, it elicits an open question whether antioxidant supplementation can reduce the risk of KSD. Specifically, modifying the diet-derived antioxidant intake is the most adaptable approach for consideration because people generally consume complex diet-derived antioxidants (e.g., carotene and vitamin C).

To assess the effects of diet-derived antioxidants on KSD risk, we first conducted an observational study using data from National Health and Nutrition Examination Survey (NHANES), which is an excellent cornerstone for nutrition monitoring among the US civilian population (16). In addition, we used Mendelian randomization (MR), a method that theoretically avoids residual confounding and reverse causality (17, 18), to further evaluate the causal relationship between lifelong diet-derived circulating antioxidant levels and the risk of KSD.

## Materials and Methods

### The Study Population in NHANES

NHANES is a cross-sectional survey, and a new data have been updated and released every two years since 1999 (16). In the present observational study, we used data from NHANES 2007–2018 since these six cycles (2007–2008, 2009–2010, 2011–2012, 2013–2014, 2015–2016, and 2017–2018, respectively), specifically inquired information about KSD. Detailed inclusion and exclusion criteria were presented in Supplementary Figure 1.

### Diet-Derived Antioxidants and Covariates

Six main diet-derived antioxidants, including retinol (a major functional compound of the vitamin A family), β-carotene (a most notable provitamin A carotenoid), vitamin B6 (an active form pyridoxal 50-phosphate), vitamin C (an ascorbate), α-tocopherol (a major molecule of the vitamin E family), and lycopene were considered in the present study. Dietary intake of these antioxidants was obtained from the total nutrient intake file, which contained summed nutrients from all the foods and beverages. All the participants were eligible for two 24 h dietary recalls, and the average consumption from two recalls would be adopted in our analysis. When any antioxidant was analyzed, the remaining five were included as covariates. Other covariates included age, gender, body weight (normal weight, overweight, or obese), race and ethnicity, education level, smoking status, hypertension (yes or no), diabetes (yes, no, or borderline), dietary intake of calcium, caffeine, sodium, potassium, vitamin D, water, protein, alcohol, and total energy according to previous studies (19, 20).

### Genome-Wide Association Studies Sources

Supplementary Table 1 demonstrates the details of genetic instruments in the present study. Briefly, we obtained single-nucleotide polymorphisms (SNPs) that were significantly associated with the exposures in published GWAS. The SNPs used for circulating retinol and α-tocopherol levels came from the same cohorts of Caucasians (*n* = 5,006) (21, 22). Genetic instruments for β-carotene were derived from another GWAS within 2,344 participants of European descent (23). As for vitamin B6, SNPs were extracted from a meta-analysis of three cohorts comprising 4,763 individuals of European descent and a MR study (24, 25). Eleven vitamin C SNPs were obtained from the largest study to date, which enrolled 52,018 European ancestry individuals (26). SNPs associated with lycopene were extracted from a GWAS conducted in 441 older Amish adults, accounting for 30.1% of the variance in circulating lycopene concentrations (27, 28). Most genetic instruments described above were commonly used in previous MR research (28, 29).

We used summary statistics for KSD from the FinnGen study, an ongoing nationwide cohort study among the Finnish, for the primary analyses (30). This GWAS consisted of 3,856 combined kidney and ureter stones and 172,757 controls. Furthermore, we conducted second analysis to validate the associations using a cohort from the UK biobank through MR base, a platform that integrates a curated database of relevant GWAS results (31, 32). This dataset is comprised of 2,427 self-reported diagnosis of kidney and ureter stone cases and 334,772 controls (33).

### Statistical Analysis

Given the complex probability cluster design of NHANES, all the statistical analyses in the present study took weights into account. Since we combined six 2-year cycles in the present research, the new weights were calculated by dividing the 2-year cycle weights by six. We categorized the intake of antioxidants into quartiles with quartile 1 as reference. We then used Stata 15.0 (Stata Corporation, College Station, TX, USA) to perform weighted multivariable-adjusted logistic regression, and *p* < 0.05 was considered statistically significant. Results were presented as the odds ratio (OR) and 95% confidence interval (CI).

The two-sample MR analyses were performed using R software. To eliminate linkage disequilibrium (LD), we pruned SNPs with the stringent pairwise *r*^2^ >0.001. However, the statistical threshold was set as 0.1 for vitamin B6 due to availability of few significant SNPs, which was a common method that had been used in MR research (Supplementary Table 1). Variance explained (*R*^2^) by genetic tools were either derived from the original study or calculated as referred to the published study (34). A calculated F-statistic was to test whether each SNP is strongly associated with exposure (34, 35). The estimate of each SNP on KSD was calculated by the Wald method (36). We calculated the total effects by combining each estimate via the inverse variance weighted (IVW) method. Additionally, the following methods were performed as sensitivity analyses; the MR-Egger (37), the weighted-mode (38), and the weighted-median method (39). MR Pleiotropy RESidual Sum and Outlier (PRESSO) were performed to determine any outlier and horizontal pleiotropy (37). We also used MR-Egger intercept to test the directional pleiotropy and Cochrane *Q* test to detect potential heterogeneity (40). Results were expressed as ORs with 95% CI on KSD risk per unit change. Supplementary Table 1 shows the detailed unit representations that μg/L in natural log-transformed scale for retinol and β-carotene, pmol/ml in natural log-transformed scale for vitamin B6, μmol/l in SD change for vitamin C, mg/L in log-transformed scale for α-tocopherol, and μg/dl for lycopene.

## Results

### Associations Between Diet-Derived Antioxidants Intake and KSD Risk in NHANES

**Figure 1:**
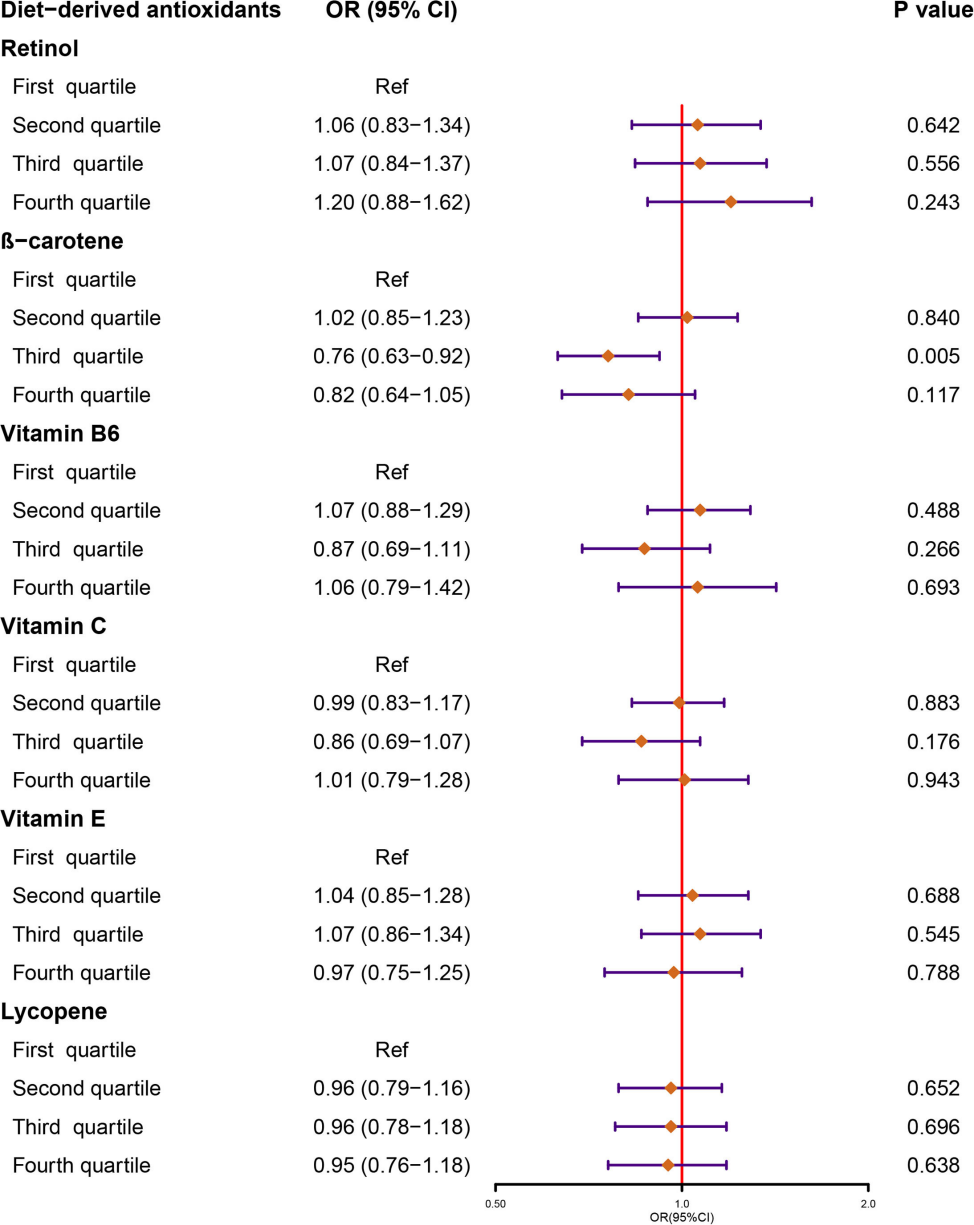
Forest plot demonstrates the summary estimates of associations between the intakes of diet-derived antioxidants, including retinol, β-carotene, vitamin B6, C, and E, and lycopene with the risk of kidney stones. ORs, odds ratios; CI, confidence interval.

Finally, NHANES 2007–2018 was comprised of six 2-year cycle data of 26,438 participants for analysis and among them, 2,543 were stone formers (Supplementary Figure 1). Results from our weighted logistic regression indicated that there were no significant associations between retinol, vitamin B6, vitamin C, vitamin E, and lycopene intake with the risk of KSD across all the quartile categories (Figure 1, Supplementary Table 2). As for β-carotene, individuals in quartile 3 had lower incidence of KSD compared to quartile 1 (OR, 0.76, 95% CI: 0.63, 0.92; *p* = 0.005). However, this inverse association was not significant in quartile 4 (OR, 0.82, 95% CI: 0.64, 1.05; *p* = 0.117).

### Causal Relationships Between Circulating Diet-Derived Antioxidants Levels and KSD Risk in MR

**Figure 2:**
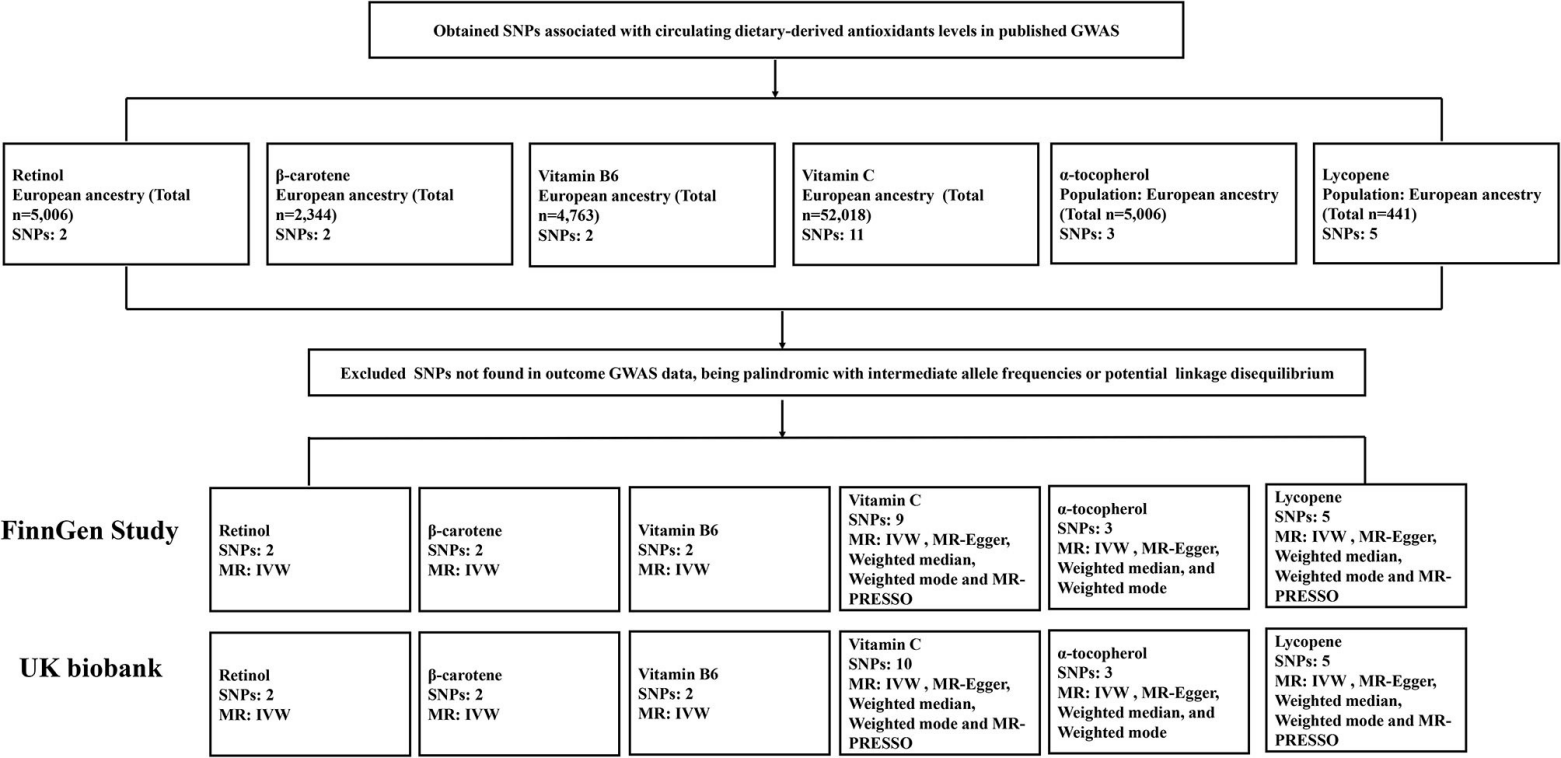
Flow chart summarizing the antioxidants studied, identification of genetic instruments, and data and MR methods used for analyses. SNPs, single-nucleotide polymorphisms; GWAS, genome-wide association studies; MR, Mendelian randomization; IVW, inverse variance weighted; MR-PRESSO, MR-Pleiotropy RESidual Sum and Outlier.

**Figure 3:**
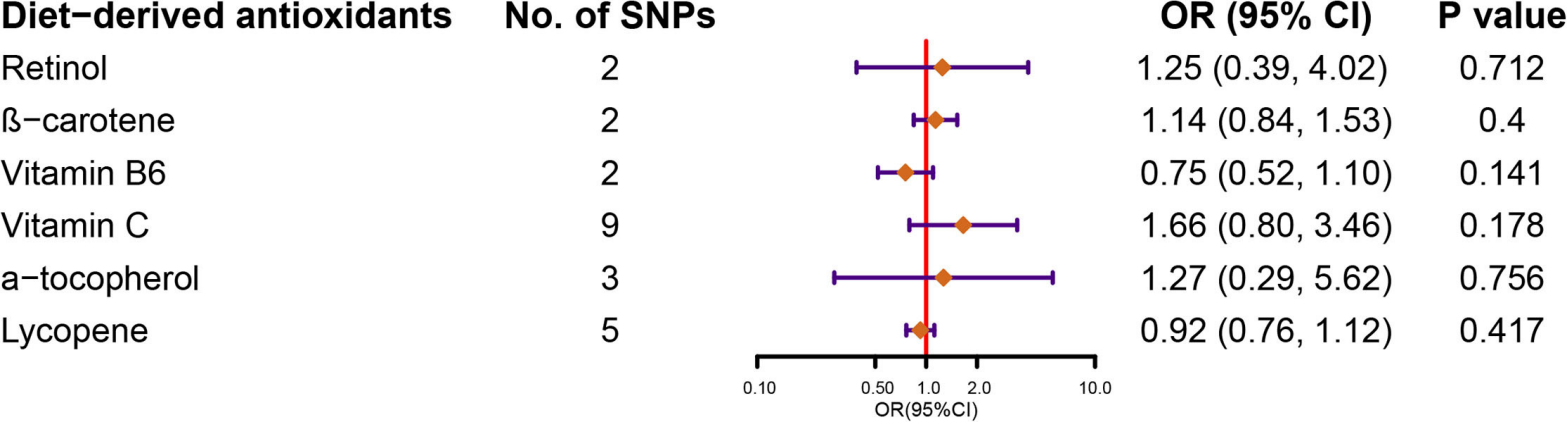
Forest plot demonstrates the summary estimates of causal relationships between circulating diet-derived antioxidants with the risk of kidney stones. SNPs, single-nucleotide polymorphisms; ORs, odds ratios; CI, confidence interval.

Figure 2 demonstrates the flow chart of identifying genetic instruments and MR methods used for analysis. Detailed summary information of these SNPs was given in Supplemental Table 3. The F-statistic for each SNP was above 10 except for β-carotene that has associated SNP rs7501331. In the primary analysis, using FinnGen study data, we did not find genetic instruments associated with circulating diet-derived antioxidants levels associated with the risk of KSD by the IVW method (Figure 3). Pooled OR for KSD risk in genetically predicted per unit change were 1.25 (95% CI: 0.39, 4.02; *p* = 0.712), 1.14 (95% CI: 0.84, 1.53; *p* = 0.400), 0.75 (95% CI: 0.52, 1.10; *p* = 0.141), 1.66 (95% CI: 0.80, 3.46; *p* = 0.178), 1.27 (95% CI: 0.29, 5.62; *p* = 0.756), and 0.92 (95% CI: 0.76, 1.12; *p* = 0.417) for retinol, β-carotene, vitamin B6, vitamin C, α-tocopherol, and lycopene, respectively. There was evidence of heterogeneity of IVW analysis for vitamin C (*p* < 0.001) and lycopene (*p* = 0.010; Supplementary Table 4).

For vitamin C, α-tocopherol, and lycopene with three or more genetic variants, we used weighted-median, weighted mode, and MR-Egger methods to conduct sensitivity analysis. Compared to IVW, the estimates did not change substantially (Supplementary Table 4). In addition, MR-Egger intercept tests suggested no evidence of pleiotropy. For vitamin C and for lycopene with five or more genetic instruments, MR-PRESSO was conducted. The estimates did not change substantially after removing outliers compared with IVW (Supplementary Table 4).

The above estimates were replicated in the secondary analysis using UK biobank data (Supplementary Table 5).

## Discussion

Our findings in NHANES suggested that the risk of KSD was not significantly associated with diet-derived antioxidants intake. Furthermore, our MR results confirmed that genetically predicted higher circulating antioxidants levels would not mitigate KSD risk. The prior effects only represent the duration of a survey period, whereas later effects are assumed to be lifelong. Lifelong exposure to circulating antioxidants could contribute to potential biological effects, even with a minor effect. Although oxidative stress was a determinant involved in calcium oxalate stone formation (10), the findings of the present study suggested that increasing diet-derived antioxidant intake to elevate circulating antioxidants levels was unlikely to result in clinical benefit for preventing KSD.

The oxidative stress seemed to be a common feature of KSD and its related comorbidities (41). A previous cross-sectional study also found that lowest quartile serum levels of β-carotene were associated with a history of kidney stones compared to the highest quartile using data from NHANES 1988–1994 of 17,695 adults (15). Although our study suggested that quartile 3 for β-carotene intake had a lower incidence of KSD than quartile 1, this inverse association was not significant in quartile 4. Furthermore, MR analyses, excluding unmeasured or residual confounding factors, demonstrated null associations between lifelong circulating β-carotene levels and KSD risk.

In the same study, serum vitamins A, E, and lycopene levels were not found to be associated with kidney stones (15) even if there was evidence that vitamins A and E could reduce crystal deposition in ethylene glycol-induced nephrolithiasis rats (42). Our study confirmed the null associations with the increased power due to a larger weighted numbers and MR design.

As for vitamins B6 and C, the associations might be more complicated to discuss because they might be associated with oxalate metabolisms beyond their antioxidant functions (43, 44). Vitamin B6 was one of the most critical molecules involved in cellular metabolism and was well-recognized as an essential antioxidant (45). Vitamin B6 supplement had also been shown to reduce oxalate excretion in urine in several studies (46, 47), but not in others (48, 49). However, not all observational studies demonstrated that vitamin B6 intake could reduce KSD risk (45, 50, 51). Recently, Ferraro et al. prospectively examined this issue in the largest-to-data cohorts, including Health Professionals Follow-up Study (HPFS) (42,919 men), Nurses' Health Study (NHS) I (60,003 older women), and NHS II (90,629 younger women) (45). Their findings were consistent with our results, suggesting vitamin B6 did not affect the risk of KSD.

Vitamin C is one of the most common antioxidants and has been known to be associated with human health (52). Nevertheless, caution should be taken because vitamin C potentially increases the risk of calcium oxalate stone formation being a precursor of oxalate (44). Studies investigating the effects of vitamin C intake on KSD risk reported conflicting results, with no association among males in the HPFS cohort (50) or older females in the NHS I cohort (51), while there was a positive association with a higher risk for KSD in males, but not in females (NHS I and II, and HPFS) (53). The lack of agreement among these observational studies may be partly due to unmeasured confounding factors and to reverse causality. Our MR results, based on general populations, expanded our understanding of this issue that lifelong higher circulating vitamin C levels were not associated with KSD risk. Therefore, elevated serum vitamin C levels caused by vitamin C intake would not increase the risk of KSD.

The major strength of our study was the use of MR analyses combined with observational study design in NHANES. The large sample size in NHANES supported us in taking many dietary factors as covariates in multivariable-adjusted logistic regression analyses simultaneously. Also, the use of MR analyses theoretically avoids potential bias factors. In addition, the consistency of findings between the MR analyses and the observational study made results more robust. Besides, we used two separate sets of KSD GWAS data and generated similar results, supporting the robustness of our MR analyses.

The present study also had some limitations. First, the limited number of SNPs for retinol, β-carotene, and vitamin B6 restricted us from performing sensitivity analyses. In addition, the relatively low variability explained by few SNP has limited the statistical power of MR analyses, even with the enormous availability of KSD sample size. Therefore, our findings should be interpreted cautiously, and identification of more SNPs associated with antioxidants through larger GWAS would improve further MR analyses. Second, we could not assess the synergistic and antagonistic interactions between two or more antioxidants using MR methods. Nonetheless, these effects might be significant in daily diets for preventing chronic diseases (54). Third, some dietary-derived antioxidants might be associated with oxalate metabolisms, such as vitamin B6 (43) and C (44). Nevertheless, we did not find evidence which can suggest their associations with KSD risk. Fourth, although no directional pleiotropy was detected in our study, there might still be some potential pleiotropies existing. Finally, differential associations between men and women in MR should be explored in the future because previous observational studies indicated that there might be disparate results by sex for vitamin C.

## Conclusions

Our study did not support that taking diet-derived antioxidants, including retinol, β-carotene, vitamin B6, C, and E, and lycopene, and that elevating lifelong circulating levels of these antioxidants are associated with KSD risk. However, these findings should be verified in larger sample-size MR due to the pleiotropy and other limitations.

## Data Availability Statement

The original contributions presented in the study are included in the article/Supplementary Material, further inquiries can be directed to the corresponding author/s.

## Ethics Statement

Ethical review and approval was not required for the study on human participants in accordance with the local legislation and institutional requirements. Written informed consent for participation was not required for this study in accordance with the national legislation and the institutional requirements.

## Author Contributions

ZJ, MW, and KW: formulating the research question. ZJ, MW, XJ, HL, and KW: designing the study. ZJ and MW: carrying out the study and writing the article. ZJ, MW, and XJ: analyzing the data. ZJ, MW, and HL: interpreting the findings. All authors contributed to the article and approved the submitted version.

## Funding

This study was supported by the National Natural Science Foundation of China, Grant/Award Nos. 81770703 and 81970602; and the 1·3·5 Project for Disciplines of Excellence, West China Hospital, Sichuan University (ZYGD18011 and ZYJC18015); and the Post-Doctor Research Project, West China Hospital, Sichuan University, Grant/Award No. 2020HXBH016.

## Conflict of Interest

The authors declare that the research was conducted in the absence of any commercial or financial relationships that could be construed as a potential conflict of interest.

## Publisher's Note

All claims expressed in this article are solely those of the authors and do not necessarily represent those of their affiliated organizations, or those of the publisher, the editors and the reviewers. Any product that may be evaluated in this article, or claim that may be made by its manufacturer, is not guaranteed or endorsed by the publisher.
