# Supplementary material for: Diet-Derived Antioxidants and Risk of Kidney Stone Disease: Results From the NHANES 2007–2018 and Mendelian Randomization Study
Source: Front Nutr. 2021 Dec 21;8:738302. doi: 10.3389/fnut.2021.738302 (PMC8724258; doi:10.3389/fnut.2021.738302)
Supplement: Supplementary file 1 [file Data_Sheet_1.docx]

Supplementary Material

**Supplementary Table 1.** Details of the genetic tools identified for circulating dietary-derived antioxidants levels

| Antioxidants | Participants (n) | LD (r^2^) | Number of SNPs before (after) LD | P value | Unit | Explained variance (R^2^)^b^ |
| --- | --- | --- | --- | --- | --- | --- |
| Retinol | 5,006 Caucasian individuals drawn from two cohorts of men: the Alpha- Tocopherol, Beta-Carotene Cancer Prevention (ATBC) Study and the Prostate, Lung, Colorectal, and Ovarian (PLCO) Cancer Screening Trial. | < 0.001 | 2 (2) | 5E-08 | µg/L in natural log-transformed scale | 2.3% |
| β-carotene | 7 case control data sets (n = 2,344) within the Nurses’ Health Study | < 0.001 | 3 (2) | 5E-08 | µg/L in natural log-transformed scale | 9.0% |
| Vitamin B6 | 4,763 individuals consisting of 1,658 women in NHSCGEMS,  1,647 women in Framingham-SNP-Health Association Resource (SHARe) and 1,458 men in SHARe | < 0.1 ^a^ | 2 (2) | 5E-08 | pmol/mL in natural log-transformed scale | 3.2% |
| Vitamin C | 52,018 individuals from the Fenland study (n = 10,771), European Prospective Investigation into Cancer and Nutrition (EPIC)-InterAct study (n = 16,841), EPIC Norfolk study (n =16,756, excluding duplicated samples with EPIC-InterAct), and the EPIC-CVD study (n = 7,650, excluding duplicated samples with EPIC-InterAct or EPIC-Norfolk). | < 0.001 | 11 (10) | 5E-08 | µmol/l in SD unit | 1.9% |
| α-tocopherol | 5,006 Caucasian individuals drawn from two cohorts of men: the Alpha- Tocopherol, Beta-Carotene Cancer Prevention (ATBC) Study and the Prostate, Lung, Colorectal, and Ovarian (PLCO) Cancer Screening Trial. | < 0.001 | 3 (3) | 5E-08 | mg/L in log-transformed scale | 1.7% |
| Lycopene | 441 Old Order Amish adults | < 0.001 | 5 (5) | 1E-06^c^ | µg/dL | 30.1% |

SNPs, single-nucleotide polymorphisms; LD, linkage disequilibrium

a Relaxing the statistical threshold for LD due to limited number of SNPs

b Variance explained (R^2^) were either derived from the original study or calculated R^2^ ≈ 2β^2^ *f*(1-*f*) [1], where β and f denote the effect estimate and the effect allele frequency of the allele on a standardized phenotype, respectively.

c Relaxing the statistical threshold for genetic instruments has been used in MR research when few significant SNPs are available.

**Supplementary Table 2.** Weighted multivariable-adjusted logistic regression of kidney stone risk for vitamin E, vitamin C, vitamin B6, retinol, lycopene, and beta-carotene intake in the 2007-2018 NHANES

|  | OR | 95% CI | | *P* value |
| --- | --- | --- | --- | --- |
|  |  | Lower limit | Upper limit |  |
| Retinol (µg/day) |  |  |  |  |
| First quartile | Ref | - | - | - |
| Second quartile | 1.06 | 0.83 | 1.34 | 0.642 |
| Third quartile | 1.07 | 0.84 | 1.37 | 0.556 |
| Fourth quartile | 1.20 | 0.88 | 1.62 | 0.243 |
| β-carotene (µg/day) |  |  |  |  |
| First quartile | Ref | - | - | - |
| Second quartile | 1.02 | 0.85 | 1.23 | 0.840 |
| Third quartile | 0.76 | 0.63 | 0.92 | 0.005 |
| Fourth quartile | 0.82 | 0.64 | 1.05 | 0.117 |
| Vitamin B6 (mg/day) |  |  |  |  |
| First quartile | Ref | - | - | - |
| Second quartile | 1.07 | 0.88 | 1.29 | 0.488 |
| Third quartile | 0.87 | 0.69 | 1.11 | 0.266 |
| Fourth quartile | 1.06 | 0.79 | 1.42 | 0.693 |
| Vitamin C (mg/day) |  |  |  |  |
| First quartile | Ref | - | - | - |
| Second quartile | 0.99 | 0.83 | 1.17 | 0.883 |
| Third quartile | 0.86 | 0.69 | 1.07 | 0.176 |
| Fourth quartile | 1.01 | 0.79 | 1.28 | 0.943 |
| Vitamin E (mg/day) |  |  |  |  |
| First quartile | Ref | - | - | - |
| Second quartile | 1.04 | 0.85 | 1.28 | 0.688 |
| Third quartile | 1.07 | 0.86 | 1.34 | 0.545 |
| Fourth quartile | 0.97 | 0.75 | 1.25 | 0.788 |
| Lycopene (µg/day) |  |  |  |  |
| First quartile | Ref | - | - | - |
| Second quartile | 0.96 | 0.79 | 1.16 | 0.652 |
| Third quartile | 0.96 | 0.78 | 1.18 | 0.696 |
| Fourth quartile | 0.95 | 0.76 | 1.18 | 0.638 |

NHANES, National Health and Nutrition Examination Survey; OR, odds ratio, CI, confidence interval

^a^ When any antioxidant was analyzed, the remaining five were included as covariates. Other covariates including age (continuous), gender (male or female), body weight (normal weight, overweight or obese), race and ethnicity (Mexican American, other Hispanic, non-Hispanic White, non-Hispanic Black, Other Race-Including Multi-Racial), education level (less than 9th grade, 9-11th grade, high school graduate/GED or equivalent, some college or AA degree, college graduate or above), smoking status (smoked at least 100 cigarettes in life or not), hypertension (yes or no), diabetes (yes, no, or borderline), and dietary intake of calcium, caffeine, sodium, potassium, vitamin D, water, protein, alcohol and total energy

**Supplementary Table 3**. Effect estimates of the associations between genetic instrumental variables for circulating dietary-derived antioxidants and risk of kidney stone disease

| Antioxidants | SNPs | Nearest gene | Effect allele | EAF | Variance (R^2^)^a^ | F statistic^b^ | Exposure | | FinnGen study | | UK biobank | |
| --- | --- | --- | --- | --- | --- | --- | --- | --- | --- | --- | --- | --- |
|  |  |  |  |  |  |  | β | se | β | se | β | se |
| Retinol | rs10882272 | RBP4 | C | 0.35 | 0.011 | 58 | -0.03 | 0.004 | 0.003 | 0.0248 | 0.000152 | 0.000212 |
| Retinol | rs1667255 | TTR | C | 0.31 | 0.011 | 58 | 0.03 | 0.004 | 0.0171 | 0.0259 | 0.000214 | 0.000211 |
| β-carotene | rs6564851 | BCMO1 | G | 0.36 | 0.010 | 24 | 0.149 | 0.02 | 0.0109 | 0.0245 | -0.0000121671 | 0.000206 |
| β-carotene | rs7501331 | BCMO1 | T | 0.24 | 0.002 | 4 | -0.067 | 0.02 | -0.0282 | 0.0257 | 0.0001 | 0.000241 |
| Vitamin B6 | rs1256335 | ALPL | A | 0.79 | 0.011 | 51 | 0.14 | 0.02 | -0.0634 | 0.0298 | -0.00073 | 0.000247 |
| Vitamin B6 | rs4654748 | NBPF3 | T | 0.52 | 0.021 | 102 | 0.1 | 0.01 | -0.0067 | 0.0239 | 1.92E-05 | 0.000206 |
| Vitamin C | rs10051765 | RGS14 | C | 0.342 | 0.001 | 35 | 0.039 | 0.007 | 0.1277 | 0.0244 | 0.00127 | 0.000218 |
| Vitamin C | rs10136000 | AKT1 | A | 0.283 | 0.001 | 32 | 0.04 | 0.007 | 0.0276 | 0.0263 | -0.00022 | 0.00023 |
| Vitamin C | rs117885456 | SNRPF | A | 0.087 | 0.001 | 45 | 0.078 | 0.012 | -0.0104 | 0.0429 | 0.000282 | 0.000364 |
| Vitamin C | rs13028225 | SLC23A3 | T | 0.857 | 0.003 | 131 | 0.102 | 0.009 | NA | NA | 0.000132 | 0.000297 |
| Vitamin C | rs174547 | FADS1 | C | 0.328 | 0.001 | 30 | 0.036 | 0.007 | 0.0248 | 0.0242 | -0.00013 | 0.000216 |
| Vitamin C | rs2559850 | CHPT1 | A | 0.598 | 0.002 | 84 | 0.058 | 0.006 | -0.0116 | 0.0243 | 0.000295 | 0.000212 |
| Vitamin C | rs33972313 | SLC23A1 | C | 0.968 | 0.008 | 407 | 0.36 | 0.018 | 0.0061 | 0.0815 | 0.00045 | 0.00056 |
| Vitamin C | rs56738967 | MAF | C | 0.321 | 0.001 | 38 | 0.041 | 0.007 | 0.0308 | 0.0258 | -0.00022 | 0.000221 |
| Vitamin C | rs6693447 | RER1 | T | 0.551 | 0.001 | 38 | 0.039 | 0.006 | -0.0107 | 0.0239 | 0.000317 | 0.000207 |
| Vitamin C | rs9895661 | BCAS3 | T | 0.817 | 0.001 | 60 | 0.063 | 0.008 | 0.1707 | 0.0308 | 0.000773 | 0.000275 |
| α-tocopherol | rs11057830 | SCARB1 | A | 0.15 | 0.003 | 17 | 0.03 | 0.01 | -0.0443 | 0.0348 | 0.000344 | 0.000299 |
| α-tocopherol | rs2108622 | CYP4F2 | T | 0.21 | 0.002 | 10 | 0.03 | 0.01 | 0.0434 | 0.0299 | 4.99E-05 | 0.000224 |
| α-tocopherol | rs964184 | BUD13/ZNF259/APOA5 | G | 0.15 | 0.002 | 10 | 0.04 | 0.01 | 0.011 | 0.0336 | -0.00063 | 0.000304 |
| Lycopene | rs2232315 | G6PC2 | A | 0.03 | 0.057 | 26 | 0.74 | 0.15 | 0.0505 | 0.1675 | -0.0006 | 0.000752 |
| Lycopene | rs341075 | ART2P | A | 0.02 | 0.061 | 28 | -0.87 | 0.17 | 0.1547 | 0.0635 | -0.00021 | 0.000607 |
| Lycopene | rs4635297 | BC039545 | A | 0.08 | 0.063 | 29 | 0.26 | 0.05 | -0.0178 | 0.031 | 0.000295 | 0.000264 |
| Lycopene | rs6108801 | C20orf187 | C | 0.04 | 0.066 | 31 | -0.48 | 0.09 | 0.1748 | 0.1049 | 0.000107 | 0.000554 |
| Lycopene | rs7680948 | SETD7 | A | 0.2 | 0.090 | 44 | -0.19 | 0.03 | -0.069 | 0.0277 | 0.0003 | 0.000232 |

SNPs, single-nucleotide polymorphisms; EAF, effect allele frequency;

a Variance explained (R^2^) were either derived from the original study or calculated R^2^ ≈ 2β^2^ *f*(1-*f*) [1], where β and f denote the effect estimate and the effect allele frequency of the allele on a standardized phenotype, respectively.

b F = 𝑅^2^(𝑁-2)/(1-𝑅^2^) [2], R is the proportion of the explained variance of the vitamin by the genetic instrument and N the sample size of the GWAS for the SNP-vitamin association; variance explained (R^2^) ≈ 2β^2^ *f*(1-*f*) [1], where β and f denote the effect estimate and the effect allele frequency of the allele on a standardized phenotype, respectively

**Supplementary Table 4.** Associations between genetically predicted circulating dietary-derived antioxidants levels and KSD risk, estimates using summary data from FinnGen study.

| **Antioxidants** | **IVW** |  | **MR-Egger** |  |  | **Weighted Median** | **Weighted mode** | **MR-PRESSO** |
| --- | --- | --- | --- | --- | --- | --- | --- | --- |
|  | **OR (95% CI)**  **(P value)** | **Q (P value)** | **OR (95% CI)**  **(P value)** | **Q (P value)** | **Intercept**  **(P value)** | **OR (95% CI)**  **(P value)** | **OR (95% CI)**  **(P value)** | **OR (95% CI)**  **(P value)** |
| Retinol | 1.247 (0.387, 4.018) (0.712) | 0.31  (0.575) | - | - | - | - | - | - |
| β-carotene | 1.136 (0.844, 1.527) (0.400) | 0.69  (0.405) | - | - | - | - | - | - |
| Vitamin B6 | 0.754 (0.518, 1.098) (0.141) | 1.45  (0.228) | - | - | - | - | - | - |
| Vitamin C | 1.658 (0.795, 3.460) (0.178) | 50.68  (< 0.001) | 0.927 (0.281, 3.060) (0.904) | 42.11  (< 0.001) | 0.045  (0.272) | 1.012 (0.694, 1.478)  (0.949) | 1.045 (0.716, 1.525)  (0.825) | 1.116 (0.784, 1.704)  (0.498)^a^ |
| α-tocopherol | 1.267 (0.286, 5.617) (0.756) | 3.66  (0.161) | 1.624 (0.374, 7.042) (0.960) | 3.65  (0.056) | -0.008  (0.979) | 1.452 (0.322, 6.543)  (0.627) | 1.625 (0.306, 8.637)  (0.626) | - |
| Lycopene | 0.924 (0.763, 1.118) (0.417) | 13.16  (0.010) | 0.736 (0.576, 0.942) (0.093) | 5.10  (0.165) | 0.098  (0.118) | 0.884 (0.763, 1.024)  (0.101) | 0.865 (0.756, 0.990)  (0.103) | 0.862 (0.777, 0.956)  (0.067)^b^ |

KSD = Kidney stone disease, IVW = Inverse variance weighted, MR = Mendelian randomization, MR-PRESSO = MR Pleiotropy RESidual Sum and Outlier.

^a^ SNPs rs10051765, rs33972313, and rs9895661 were tested as outliers.

^b^ SNPs rs7680948 was tested as outlier.

**Supplementary Table 5.** Associations between genetically predicted circulating dietary-derived antioxidants levels and KSD risk, estimates using summary data from UK biobank.

| **Antioxidants** | **IVW** |  | **MR-Egger** |  |  | **Weighted Median** | **Weighted mode** | **MR-PRESSO** |
| --- | --- | --- | --- | --- | --- | --- | --- | --- |
|  | **OR (95% CI)**  **(P value)** | **Q (P value)** | **OR (95% CI)**  **(P value)** | **Q (P value)** | **Intercept**  **(P value)** | **OR (95% CI)**  **(P value)** | **OR (95% CI)**  **(P value)** | **OR (95% CI)**  **(P value)** |
| Retinol | 1.001 (0.989, 1.013) (0.863) | 1.50  (0.221) | - | - | - | - | - | - |
| β-carotene | 1.000 (0.997, 1.002) (0.838) | 0.14  (0.714) | - | - | - | - | - | - |
| Vitamin B6 | 0.997 (0.992, 1.002) (0.274) | 3.93  (0.047) | - | - | - | - | - | - |
| Vitamin C | 1.003 (0.999, 1.008) (0.168) | 41.06  (< 0.001) | 1.001 (0.994, 1.008) (0.853) | 37.39  (< 0.001) | 0.0002  (0.401) | 1.001 (0.999, 1.004) (0.359) | 1.001 (0.999, 1.004) (0.239)) | 1.002 (0.999, 1.005) (0.173)^a^ |
| α-tocopherol | 0.997 (0.982, 1.012) (0.715) | 5.30  (0.071) | 0.925 (0.863, 0.991)  (0.269) | 0.63  (0.430) | 0.003  (0.276) | 0.999 (0.984, 1.014)  (0.890) | 1.002 (0.984, 1.020)  (0.867) | - |
| Lycopene | 1.000 (0.999, 1.001) (0.854) | 3.68  (0.451) | 1.000 (0.998, 1.002)  (0.866) | 3.55  (0.314) | -0.0001  (0.766) | 1.000 (0.999, 1.001)  (0.993) | 1.000 (0.991, 1.001)  (0.890) | None outliers |

KSD = Kidney stone disease, IVW = Inverse variance weighted, MR = Mendelian randomization.

^a^ SNPs rs10051765 was tested as outlier.

References

[1] Park JH, Wacholder S, Gail MH, Peters U, Jacobs KB, Chanock SJ, et al. Estimation of effect size distribution from genome-wide association studies and implications for future discoveries. Nat Genet. 2010;42:570-5.

[2] Burgess S, Thompson SG, Collaboration CCG. Avoiding bias from weak instruments in Mendelian randomization studies. Int J Epidemiol. 2011;40:755-64.

**
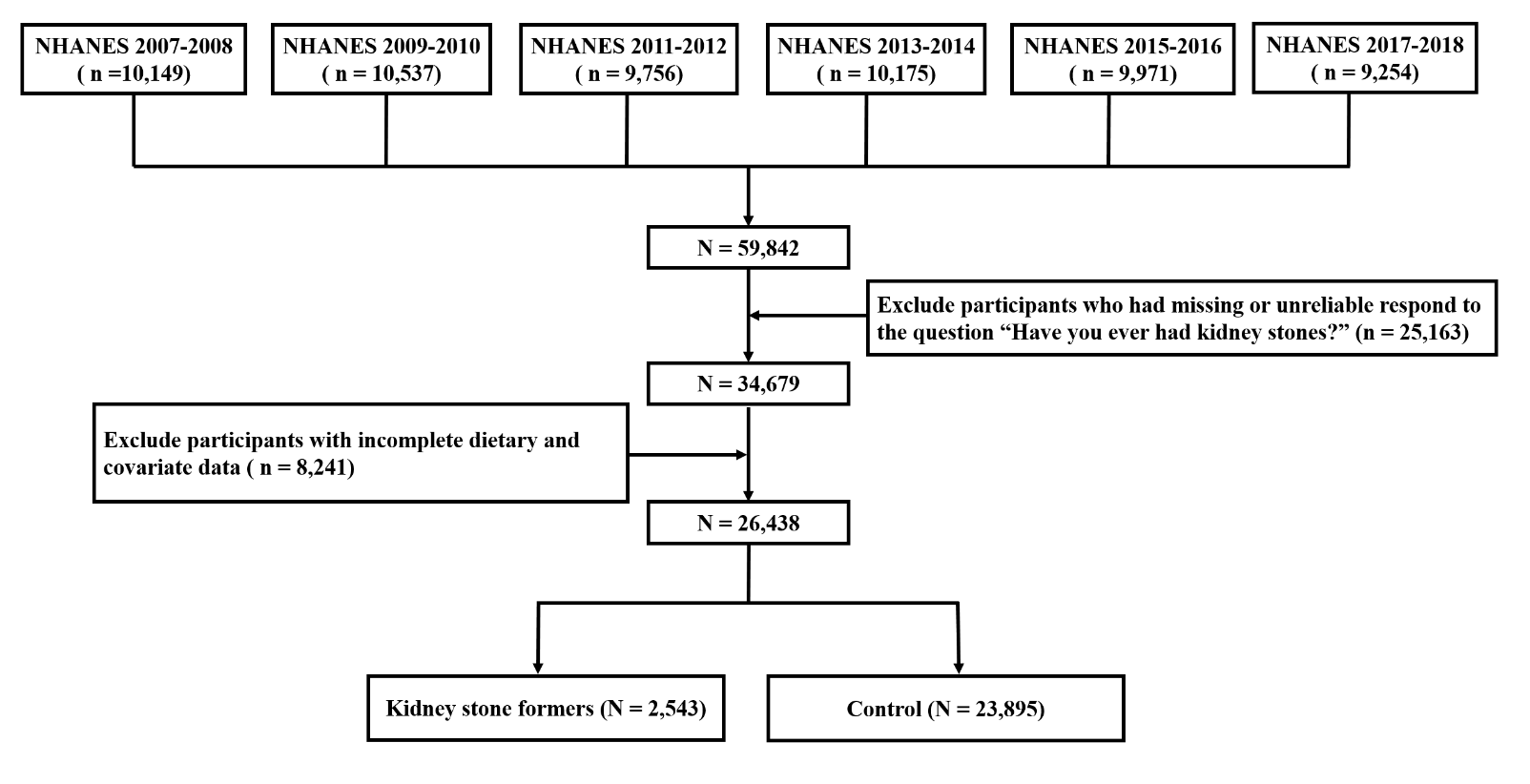
Supplementary Figure 1.** Flow chart of selection process for NHANES 2007-2018
